# Arsenic Eating

**Published:** 1865-09

**Authors:** 


### ARSENIC EATING.

Dr. Maclagan, of Edinburgh, on a visit to Styria in the spring of this year, obtained conclusive evidence of the existence of this practice, and has published in the *Edinburgh Medical Journal* a circumstantial account of what he saw. We quote one case, in which it will be seen no jugglery could have been practised: "Mathias Schober, a healthy-looking, fresh-complexioned, fairly muscular young man, of the age of 26 years, and about five feet nine inches in height, a native of Liegest, and employed as a house servant there, said he had taken Huttereich for about a year and a half—not, however, with arsenic, but the yellow arsenic, or orpiment, of which he took a specimen from his pocket and showed it to me. Of this I retained a piece for chemical investigation. He informed me that he took the arsenic in order to keep strong, though he had never suffered from ill health. He said he had never experienced any bad effects, even when he first began using it; that he had at first taken rather less than a grain every fortnight; that he now took it twice a week; and that on omitting to take it for any longer period he experienced a longing for it, which was relieved by a repition of the usual dose. His reason for taking the orpiment instead of white arsenic was, that it was more easily procured; but having professed himself quite indifferent whether it were arsenious acid or the sulphuret, Dr. Knappe produced a paper containing the former (of which I also kept a sample), and having asked him to choose out a piece such as he was in the habit of taking, it was weighed, and found to be nearly five grains. We had no finer weight than one grain; but the piece of arsenic was much over four, though less than five. Dr. Knappe, having carefully ground this to powder on a clean piece paper, it was transferred to a small piece of plain white bread, about as large as a man's thumb-nail, and this the doctor put into his mouth. Schober chewed it and swallowed it, and then swallowed another portion of bread the same size immediately after. This was at 9:30 A. M. He stayed with us a few minutes, but he had to return to his work, promising, however, to come back in a short while.

This he did at 11:30, two hours after, and made water in my presence to the amount of what I estimated at twenty-eight ounces, into a vessel previously carefully cleaned, and the urine was put into bottles thoroughly washed by myself. Unfortunately, in the hurry of my departure, in trying to pack these bottles into my hat-box, I broke one, and thus lost part of the urine. Since my arrival in this country I subjected the contents of the two remaining bottles to chemical analysis, adopting the distillation process of Dr. Taylor as the most convenient way of separating arsenic from the organic matters of the urine. For this purpose the urine was carefully evaporated to dryness in a clean retort. The nearly dry residue was covered with strong hydrochloric acid, and distilled into a well-cooled receiver. The product, amounting to about half an ounce, was a clear, feebly pinkish fluid, thirty minims of which, when treated both by Reinsch's and Marsh's process, gave very characteristic arsenical deposits. Schober also came the following day to see me, having taken no more arsenic since the dose which he had swallowed before me twenty-six hours previously. I again secured some urine which he passed in my presence, and this, when chemically examined as above, also yielded arsenic freely." Mr. Maclagan adds—" It is evident that the confirmation of the existence of the practice of arsenic-eating must lead us to modify some of the opinions that are entertained with regard to the influence of habit on the action of poisons. It has long been notorious that, by habit, the human body may be brought to bear with impunity doses of organic poisons, such as opium, which, to those unaccustomed to them, would certainly prove fatal; but 'it has hitherto been considered by toxicologists that, except within very narrow limits, habit appears to exercise no influence on the action of mineral poisons.' (Taylor "On Poisons," p. 89.) Though the experiment of M. Flandin, by which he proved that he could bring dogs to bear fifteen grains of arsenious acid in powder in twenty-four hours without injury to their appetite or health, and the practice of administering arsenic to horses, have long been known as pointing rather in the contrary direction, this has been supposed to be due to some peculiarity in the constitution of the lower animals. The facts which have been ascertained with regard to the Styrian arsenic-eaters, and which the above observations confirm, entitle us to maintain that the modifying effect of habit is not confined to organic poisons, but extends to those of mineral nature—at all events, to arsenic." —*Chem. News*, July 21, 1865.

